# Relative Age Effect is Modulated by Playing Position but is Not Related to Competitive Success in Elite Under-19 Handball Athletes

**DOI:** 10.3390/sports7040091

**Published:** 2019-04-19

**Authors:** Fabiano S. Fonseca, Lucas S. Figueiredo, Petrus Gantois, Dalton de Lima-Junior, Leonardo S. Fortes

**Affiliations:** 1 Department of Physical Education, Universidade Federal Rural de Pernambuco (UFRPE), Recife 52171-900, Brazil; 2 Department of Physical Education, Faculdade de Ensino de Minas Gerais (FACEMG), Belo Horizonte 31515-000, Brazil; savassi88@hotmail.com; 3 Associated Graduate Program in Physical Education, Universidade Federal da Paraíba (UFPB), João Pessoa 58051-900, Brazil; pgm.gantois@gmail.com; 4 Graduate Program in Physical Education, Universidade Federal de Pernambuco (UFPE), Recife 50670-901, Brazil; limajunior.dalton@gmail.com; 5 Department of Physical Education, Universidade Federal da Paraíba (UFPB), João Pessoa 58051-900, Brazil; leodesousafortes@hotmail.com

**Keywords:** relative age effect, talent identification, athlete development, youth sport, handball

## Abstract

This study aimed to verify the occurrence of the relative age effect (RAE) in male elite young handball athletes according to the playing position and its association with team performance in a World Championship. Data from 383 handball athletes from 24 countries who participated in the 7^th^ World Men’s Championship in the under-19 category were analyzed. RAE was investigated from the birth trimester of the athletes, their playing position, and final ranking in the Championship. The results showed an overrepresentation of athletes born in the first two trimesters (Q1 and Q2) (χ^2^_(3)_ = 32.97; p < 0.001, ω = 0.29). The analysis of the athlete’s position showed that most wings (χ^2^_(3)_ = 18.37; p < 0.001, ω = 0.32) and backs (χ^2^_(3)_ = 12.51; p = 0.006, ω = 0.34) were born in the first trimesters (Q1 and Q2). The ranking in the Championship presented no significant association with the date of the birth (p > 0.05). The results showed the existence of the RAE in youth handball elite athletes, especially for the back and wing positions. However, the strategy of selecting is questionable once the presence of RAE was not associated with competitive success.

## 1. Introduction

In a sports context related to young athletes, annual age-grouping is a common strategy widely used to divide athletes into categories and competitive levels based on their year of birth [1]. Although the main purpose of this strategy is promoting equality, differences between those born immediately after and those born immediately before the date used to group the athletes into cohorts might cause a phenomenon known as relative age effect (RAE) [2]. RAE provides an immediate competitive advantage or in long-term sports participation of young athletes as a result of maturational and developmental processes associated with differences in chronological age [1]. With this in mind, even with the same chronological age, athletes born in the first months of the year (January–March) may present competitive advantages compared to those born in the last months (October–December). This happens because physical attributes, such as greater height and mass (to a certain degree), are crucial aspects to select athletes in sports, especially the ones that require power, speed, and endurance from athletes [3]. Thus, athletes born in the first months when compared with athletes born later in the same year may have maturational advantages that affect the most common indicators used in the selection process [4]. The occurrence of RAE may have numerous implications in the process of selection and development of young athletes, such as favoring a discriminatory effect by disadvantaging relatively younger athletes reducing their chances of achieving competitive levels and athletic development [5,6]. 

Wattie et al. [2] proposed a theoretical model to explain the existence of RAE in sports. According to this model, the emergence of RAE is explained by the interaction between three types of constraints: individual constraints, task constraints, and environmental constraints. Individual constraints include biological factors related to functional and structural characteristics (e.g., sex, height, weight, maturational status). Environmental constraints refer to the social context of the young athlete, including the physical and sociocultural environment and the influence of coaches, parents, and friends. Finally, task constraints are related to the specific demands of sports, that is, type, level of competition, physical capabilities (e.g., strength, speed, agility, flexibility, endurance), and technical-tactical abilities that are determinants for success. Together, different factors related to these three types of constraints seem to contribute varying degrees of relevance to the occurrence of the RAE in sports. Wattie’s theoretical model makes it possible to generate questions and hypotheses to analyze RAE in an integrated way to understand the phenomenon.

Relative age presents different effects depending on the characteristics of each sport [2,7]. According to Wattie’s tridimensional model, task constraints, such as physical requirements and specificities of playing position determine the occurrence of RAE [8,9]. Thus, physical requirements and specificities of playing position should be considered in team sports’ investigations. Handball is an intermittent invasion sport that requires high-intensity actions such as running, accelerations, decelerations, change of directions, jumps, throwing, and body contact demands [10]. Indeed, it has been characterized as a sport with high technical, tactical, cognitive, and physical requirements [10,11,12]. Moreover, the actions performed by athletes in different playing positions require distinct skill demands [10,11,12]. Thus, physical (e.g., strength, speed, and endurance), cognitive (e.g., decision-making performance), and anthropometric attributes (e.g., height) are individual constraints used by coaches to select and define playing positions [13]. In fact, evidence shows such characteristics favor the occurrence of RAE in handball, and playing position act as a constraint to the phenomenon [8,14]. In handball, specific attacking positions are wings, backs, pivots, and goalkeepers. The wing players generally are the smallest players and have a major participation in counter-attack actions [11]. Back players are usually the ones who perform more shoots during a match, and their height is determinant to succeed in throwing over defense [14]. The pivots, in turn, play infiltrated in the defense and are more susceptible to high-intensity contacts and clasping actions. Goalkeepers have a very specific profile, covering small distances during games and focused on reactivity and specific rapid movements [14]. 

The occurrence of RAE has been widely reported in sports where physical and anthropometrics attributes are important to performance, such as soccer [15,16], basketball [17,18], and ice hockey [19]. Regarding handball, studies are scarce, especially involving young world-class athletes and analyzing the phenomenon according to the playing position [8]. Most of the studies that investigated the presence of RAE in young elite athletes are limited since they have analyzed the phenomenon at the national level, such as France [20], Spain [21], Norway [22], Denmark [23], and Germany [8]. In general, the findings have confirmed the presence of RAE in handball and the hypothesis that playing position acts as a constraint to the RAE [8,14].

Although RAE is widely investigated in sport, most studies have been conducted primarily to describe their presence and associated factors. In basketball, it has been reported that having older athletes in U16 and U18 may not be decisive for the best performance of teams in championships [18]. This fact raises some questions, for example, to what extent the RAE is associated with competitive success, in fact, that makes questionable the current model used in the selection of young athletes for team sports. The elucidation of this question has relevant practical implications on the process of detection, selection, and development of young athletes. Thus, to the best of our knowledge, no study in handball has investigated whether RAE is associated with successful performance in competition. In the case of international handball championship relatively little information is available about RAE. This is important because it allows us to investigate if there is any advantage in selecting athletes according to the month of birth, specifically in those elite national handball athletes. Moreover, the results of the present study may indicate, even indirectly, whether to divide the base categories by chronological age is, in fact, an interesting strategy in the process of athlete development. 

Therefore, the aims of the present study were to verify the existence of RAE in young elite male handball athletes and to analyze if its occurrence is specified by the playing position and whether RAE is associated with successful team performance. Due to the characteristics and inherent demands of handball, we expected to find an RAE in young elite athletes (U-19). We also hypothesized that a large RAE would be revealed for the back position because physical size and strength are important determinants of success for specific actions in that function [8], thus, young athletes with the most advanced maturation status, physical attributes, and anthropometric measures are selected for this position [24]. Lastly, considering that competitive success in team sports is multifactorial in nature [25], that is, it depends on the interaction between several factors, such as physical, technical, tactical, and psychological components, we assume that there will be no association between RAE and success in competition.

## 2. Materials and Methods

### 2.1. Participants

The sample was composed of 393 athletes of the 7^th^ Handball Men’s Youth World Championship in the under-19 category (age 18.3 ± 0.7 years). A total of 24 countries (Algeria, Argentina, Bahrain, Brazil, Chile, Croatia, Denmark, Egypt, France, Georgia, Germany, Iceland, Japan, Korea, Mexico, Norway, Poland, Portugal, Russia, Serbia, Slovenia, Spain, Sweden, and Tunisia) participated in the Championship organized by the International Handball Federation in Georgia in 2017.

### 2.2. Data Collection and Procedures

Data were obtained from the official results book available from International Handball Federation on its webpage (http://www.ihf.info/). The book contains information about the results of the 7^th^ Handball Men’s Youth World Championship in the under-19 category, team ranking, and team roster athletes (name, position, birth date, age, and so on). The information was used to verify the existence of RAE between the athletes to compare playing positions and analyze its association with the final team ranking in the Championship.

The cut-off date for youth categories of national’s teams participating in the Handball Men’s Youth World Championship was January 1^st^ (quarters are composed as a function of this cut-off). The variables analyzed included the birth quarter of the athletes, that is, quarters of the year the athletes were born: Q1 (January, February, March), Q2 (April, May, June), Q3 (July, August, September) and Q4 (October, November, December), the specific playing position of each athlete (wings, backs, pivots, and goalkeepers), and the team’s ranking in the championship (first to last position).

### 2.3. Statistical Analysis

Data was presented in absolute and relative frequency. The birth-date distribution of the athletes was compared performing a chi-square test (χ^2^) of one variable (e.g., adopting absolute frequencies) to describe the RAE of the participants and X^2^ 2x2 test to analyze the association between the specific playing position and team performance. For all analyses, the effect size ω of the Chi-square tests was calculated according to Cobley et al. [1]. The number of athletes in each quarter was compared with the expected frequency [26]. Also, odds ratio (ORs) and 95% confidence interval were calculated for both quarter and half year’s distribution according to the team ranking (semi-finalist vs. quarter-finalists vs. octaves-finalist vs. bottom eight). The analysis was performed in the statistical package for the social sciences (SPSS) 20.0 version (Chicago, USA). Statistical significance was set at 5%.

## 3. Results

### 3.1. Birthdate Distribution by Quarters

**Figure 1:**
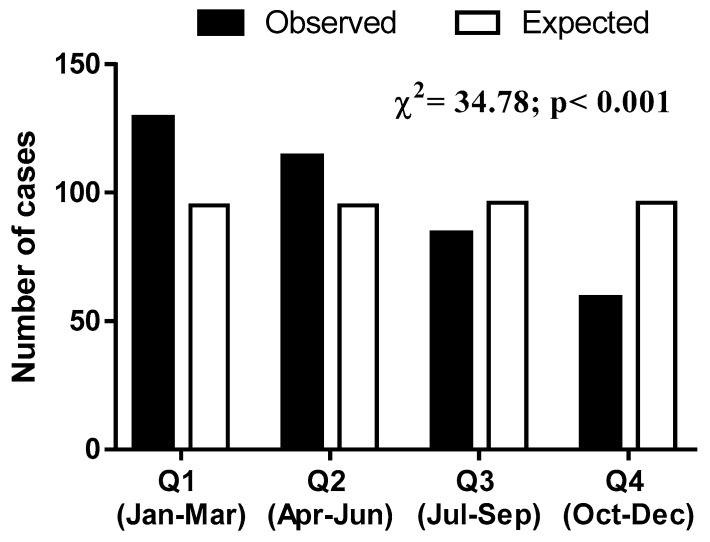
Distribution of the quarter of birth (Under-19 World Men’s Handball Championship).

Figure 1 shows the distribution in quarters of the date of birth of the handball athletes. The observed distribution was different from expected (χ^2^_(3)_ = 34.78; p < 0.001, ω = 0.30), with an overrepresentation of athletes born in the first semester (Q1 and Q2).

### 3.2. RAE vs. Player Position

The observed distribution was different according to the player’s position (Table 1). Specifically, most wings (χ^2^_(3)_ = 19.19; p < 0.001, ω = 0.33) and backs (χ^2^_(3)_ = 13.04; p = 0.005, ω = 0.37) were born in the first quarter. Conversely, the distribution of birth dates for the pivots and goalkeepers were evenly distributed (p > 0.05). 

### 3.3. RAE vs. Team Performance

Athletes born in the Q1 and Q2 were overrepresented in the quarter-finalists (χ^2^_(3)_ = 8.02; p = 0.042, ω = 0.35), octaves-finalists (χ^2^_(3)_ = 20.28; p < 0.001, ω = 0.40), and bottom eight teams (χ^2^_(3)_ = 11.89; p = 0.008, ω = 0.30) (Table 2). Birthdates were evenly distributed among the semi-finalists (χ^2^_(3)_ = 1.73; p = 0.629, ω = 0.16).

OR results are presented in Table 3. The ranking in the Under-19 World Men’s Handball Championship 2017 presented no significant association with the date of the birth (i.e., quarter and half year) (p > 0.05).

## 4. Discussion

The aim of the present study was to verify the RAE in male handball athletes in the Under-19 World Men’s Handball Championship 2017 according to the playing position and team performance. The findings showed a skewed distribution of the birth dates in Under-19 handball athletes. Specifically, more athletes were born in the first two quarters of the year. A similar pattern was observed according to the playing position for the wings and backs. On the other hand, pivots and goalkeepers were more evenly distributed. Our results also showed that despite the RAE that was observed, considering all athletes by playing position, RAE was not associated with team performance (i.e., final ranking). To the best of our knowledge, this is the first study to demonstrate a lack of association between RAE and competitive success in young elite handball athletes.

As hypothesized, RAE was verified for the back position, corroborating previous studies with young athletes [8,14]. The effect of RAE on back position is likely due to the position-specific requirements of these players. Among other actions, back athletes perform more shooting actions over defense than other line athletes, which are favored by bigger body sizes [27]. In addition, back and wings have been associated with higher strength levels [28] and throwing velocity (Krueger et al., 2014) when compared to pivots and goalkeepers. These aspects may be determinant for coaches to select athletes, leading to a preference for relatively older athletes, who may have matured in advance [24]. In the relatively older athletes, the possibility of early maturation could lead precisely to greater heights and levels of strength and speed at the time of selection [13]. The specific position demands or task constraints in Wattie’s model [2] directly interact with the characteristics of the athletes or individual constraints to explain the effect found in youth backs.

The position analysis also showed RAE for the athletes who played at the wings. Different from the backs, who are characterized by bigger and stronger bodies, the wings are usually the smaller athletes in the team [28,29], as well as being involved in more transitions between defense and attack during the game [14]. Compared to other line athletes it was found that wings cover higher distances per minute than pivots [30] and more high-intensity runs than backs and pivots [31], which requires these athletes to have greater speed and repeated-sprint abilities. Kruger at al. [29] showed that German professional wings and backs outperformed goalkeepers and pivots on a 30 m sprint test, an activity that resembles the fast transition from offense to defense and for counter attacks, which is another important part of the game in which wings and backs have greater participation [14]. Throwing velocities, jumping ability and heart rates are other variables in which higher values were found for wings and backs [29]. These findings, along with the RAE observed for wings and backs in the present study may indicate that player selection in the youth elite handball teams may have been influenced by the athlete’s physical characteristics. Faster and more resistant athletes have advantages in the wing position once the success rate of those high-intensity actions can be decisive in the outcomes. Again, the interaction between task and individual constraints explain the RAE found for the wing position [2].

Regarding performance, we found an even distribution of the birth date for the semi-finalist teams, whereas in the other teams was observed an overrepresentation of athletes born in the first months of the year. This bias was already presented at lowest level participation in handball athletes with a decreasing effect over time, indicating that relatively younger athletes still manage to achieve a higher level of competition in the sports development system [8]. The reduction of RAE in the semi-finalist teams of the Under-19 World Men’s Handball Championship 2017 is difficult to explain since several underlying mechanisms might play a role in it. The data suggest that the RAE bias is reduced in older categories and may disappear in the senior-elite stage [1,32]. The “reversal of advantage” [32] or the “underdog effect” hypotheses [33] may explain this pattern when investigating U-19 athletes. Previous studies involving different team sports demonstrated that award-winning athletes [34] with higher salaries [35], mature age draftees [36], and Canadian National Hockey League All-star athletes [33] were more likely to be born late in the year. Taken together, these data suggest that a “reversal of advantage” exist, where some athletes can increase their competitiveness and overcome their chronological limitations by developing skills related to sports context (i.e., technical and tactical performance) to compete with their pairs who were born earlier in the year [3,17]. Noteworthy, data regarding this premise is limited at present, thus, future investigations are warranted to elucidate this issue. However, the higher proportions of late-born rugby and cricket athletes who reach the senior national squads are consistent with this second hypothesis [37].

In handball, young athletes perform better according to their anthropometrics and physiological characteristics [38,39]. RAE studies showed that young athletes born earlier in the year (i.e., first semester) have an advantage in the anthropometric, physiological, technical, and tactical aspects [17,18,40]. As performance demands of handball favor the athletes with those better-developed characteristics, it is not surprising that increased selection opportunities exist for the relatively older athletes. However, many of the qualities that distinguish the successful player at adulthood may not be achieved until late adolescence [18]. Thereby, there is still a debate on how those athletes born early might get any benefit from a performance standpoint in the national team, mostly in the increased age-grouped categories (i.e., >18 years).

In the teams of the Under-19 World Men’s Handball Championship 2017, we observed that RAE was not associated with final placement in the championship. Despite the skewed birth date distribution, teams with athletes born early in the year were not likely to perform better at the championship. Additionally, previous studies showed that RAE does not predict high-performance, selection for the national team, match outcome as well as it is not presented in some Olympic sports [33,41,42,43]. Taken together, these data do not support the efficiency of the RAE since this negative selection bias in the early stage of development does not explain long-term sports achievements. Although relative age should have little to no influence on the results of team sports with higher competitiveness level and age categories (>18 years), the physical maturity differences during adolescence may explain the RAE presence on lower categories in team sports [2]. Nevertheless, this bias may represent a significant loss of potential in young handballers, also coaches within sports development systems should aim the long-term player development rather than seeking immediate performance in young athletes.

One of the primary explanations for the RAE in sport is the role of physical maturation (i.e., maturation-selection hypothesis) [1]. For instance, even in the same categories, the maturational difference tends to be reinforced according to the annually age-grouped cohorts that some athletes are born in January and others in December (i.e., almost one year later). In team sports, there is a negative bias to select young athletes born at the end of the year, since they might be less mature and have anthropometric, physiological, and physical disadvantages than their peers born in the first months of the year [2,15,44]. Although the relative age phenomenon exists, its advantage effects tend to disappear when the age category and competition level increases [3,45]. Previous studies showed that late-maturing athletes present an increased progression in anthropometric and physical attributes and “catch-up” the early-maturing athletes during adolescence and at adulthood, thus, it is likely that the later ones may overtake their peers [46,47]. Although we did not access maturity indicators in the present study, these data may support the lack of association between RAE and performance in the present study. It is possible that any previous positive RAE gradually diminished in the Under-19 categories (i.e., ~18 years) when the growth process is supposedly finished. However, in order to understand how maturity and skill level of the athletes are affected by RAE, complementary tests, such as individual evaluations of performance and specific skills could be measured, which is a limitation of the present study. To provide more information about the RAE in young elite male handball athletes, future studies should evaluate those variables. 

From a practical perspective, the results of the present study suggest that the RAE is ineffective in explaining the competitive success of national teams with young elite handball athletes. Thus, coaches are encouraged to reduce the role of the RAE on the early stages of selection processes of their athletes. Noteworthy, the only performance indicator investigated in the present study was the final ranking in the Under-19 World Men’s Handball Championship 2017. Thus, we consider the lack of other indicators (i.e., technical-tactical aspects, decision-making) a limitation of the present study. Future studies are warranted to investigate the RAE on these performance indicators. However, the scientific literature has not supported the efficiency of the RAE at senior and professional level team national athletes [1,34,36]. Long-term player development should be the major aim in the sports development system rather than an immediate match and victory in competitions. The selection process based on RAE phenomenon may represent an exclusion of a potential talented handball player due to their chronological limitation. In addition, youngest athletes are more likely to drop out from the sport, since relatively older selected athletes could benefit from more skilled coaches and higher teams and competitions levels, which in turn may increase the RAE advantages compared with those athletes born later in the year (i.e., the Matthew effect) [48]. To avoid that bias selection, the current literature state that counter-RAE interventions are warranted. Rada et al. [49] suggest that competitions should be designed without official ranking until the late adolescence, at least 40% of athletes should be born in the second half of the year, and the age-group cohort should be reduced from 12 to 9–6 months. In addition, RAE could be reduced through the employment of age-ordered shirt numbering during the selection process in order to make coaches aware of the athlete’s age [50] and observe performance considering birth distributions [51]. These cautions may reduce the maturational advantage during the adolescence and decrease the RAE at the early sports development process. 

## 5. Conclusions

In conclusion, we verified the relative age effect in young elite handball athletes. Despite the tendency of RAE disappear as age increases, our findings evidence the permanence of this phenomenon even in an age group considered advanced. The process of selecting young elite athletes in handball seems to favor those born in the first half of the year. In addition, specific demands of playing position appear to act as a task constraint and affect the magnitude of RAE. Significantly larger RAE were observed for backs and wings compared with goalkeepers and pivots. Despite the RAE occurrence in young athletes’ selection, the supposed advantage of older athletes does not seem to be a preponderant factor for competitive success in team sports. We found no association between an overrepresentation of the athletes born in the first semester and the classification of the team in championships. These findings bring the current process of athlete selection for young age groups into question. Federations, clubs, and coaches should consider that RAE might be irrelevant for high performance and reflect on alternatives to avoid the exclusion of a potentially talented handball player due to a chronological limitation.

## Figures and Tables

**Table 1:** Distribution of the quarter of births according to the player’s position (Under-19 World Men’s Handball Championship).

Quarter of Birth	Wings		Backs		Pivots		Goalkeepers	
	Obs	Exp	Obs	Exp	Obs	Exp	Obs	Exp
Q1	60 (34.1%)	43.5	32 (34.4%)	23.0	18 (30.0%)	14.8	19 (35.2%)	13.3
Q2	52 (29.5%)	43.8	31 (33.3%)	23.2	18 (30.0%)	14.9	13 (24.1%)	13.4
Q3	42 (23.9%)	44.4	18 (19.4%)	23.4	10 (16.7%)	15.1	14 (25.9%)	13.6
Q4	22 (12.5%)	44.4	12 (12.9%)	23.4	14 (23.3%)	15..1	08 (14.8%)	13.6
χ2	19.19		13.04		3.12		4.74	
ω	0.33		0.37		0.22		0.29	
Sig.	<0.001		0.005		0.373		0.192	

Q1–Q4 represents birth quarter; χ2 represents chi-square value; Obs represents Observed; Exp represents Expected; Sig. represents significance.

**Table 2:** Distribution of the quarter of births according to the ranking in the Under-19 World Men’s Handball Championship 2017.

Quarter of Birth	Semi-Finalists		Quarter-Finalists		Octaves-Finalists		Bottom Eight	
	Obs	Exp	Obs	Exp	Obs	Exp	Obs	Exp
Q1	17 (26.6%)	15.8	20 (31.3%)	15.9	48 (37.8%)	31.4	44 (34.4%)	31.9
Q2	19 (29.7%)	15.9	23 (35.9%)	15.9	34 (26.8%)	31.6	38 (29.7%)	31.9
Q3	16 (25.0%)	16.1	10 (15.6%)	16.1	32 (25.2%)	32	26 (20.3%)	32.3
Q4	12 (18.8%)	16.1	11 (17.2%)	16.1	13 (10.2%)	32	20 (15.6%)	32.3
χ2	1.73		8.02		20.28		11.89	
ω	0.16		0.35		0.40		0.30	
Sig.	0.629		0.042		<0.001		0.008	

Q1–Q4 represents birth quarter; χ2 represents chi-square value; Obs represents Observed; Exp represents Expected; Sig. represents significance.

**Table 3:** Odds ratio according to the team’s ranking in the Under-19 World Men’s Handball Championship 2017 examining relative age effect.

Ranking	ORs Comparison (95% CI)							
	Q1 vs. Q4	Sig.	Q2 vs. Q4	Sig.	Q3 vs. Q4	Sig.	1st vs. 2st	Sig.
Semi-finalists **	-		-		-		-	
**Quarter-finalists**	1.28 (0.45 to 3.64)	0.64	1.32 (0.48 to 3.66)	0.59	0.68 (0.22 to 2.13)	0.52	1.59 (0.78 to 3.3)	0.32
**Octaves-finalists**	2.61 (0.99 to 6.81)	0.06	1.65 (0.63 to 4.33)	0.30	1.85 (0.69 to 4.96)	0.22	1.41 (0.77 to 2.61)	0.26
**Bottom eight**	1.55 (0.63 to 3.85)	0.34	1.20 (0.49 to 2.96)	0.69	0.97 (0.38 to 2.52)	0.96	1.39 (0.75 to 2.56)	0.29

Q1–Q4 represents birth quarter; 1st represents first semester; 2st represents second semester; ORs represents Odds ratio; ** represents reference category.
